# The portal of Neviaser: a valid option for antegrade nailing of humerus fractures

**DOI:** 10.1186/s40634-020-00222-0

**Published:** 2020-02-29

**Authors:** Torsten Gerich, Caroline Mouton, Lea Jabbarian, Jean-Paul Weydert, Alexander Hoffmann, Dietrich Pape, Romain Seil

**Affiliations:** 1 Department of Orthopaedic Trauma, Centre Hospitalier de Luxembourg, 4, rue Barble, L-1210 Luxembourg, Luxembourg; 2 Department of Orthopaedic Surgery, Centre Hospitalier de Luxembourg, 76 rue d’Eich, L-1460 Luxembourg, Luxembourg; 3 Department of Public Health, Erasmus MC, P.O. Box 2040, Rotterdam, 3000 CA Netherlands; 4 Department of Physiotherapy, Centre Hospitalier de Luxembourg, 4, rue Barble, L-1210 Luxembourg, Luxembourg

**Keywords:** Retrospective, Non-randomized, Humerus fracture, Straight nail, Neviaser, Rotator cuff, Supraspinatus

## Abstract

**Introduction:**

The objective of this retrospective non-randomized study was to evaluate the portal of Neviaser (PN) as an alternative approach in antegrade humeral nailing.

**Methods:**

The surgical approach for the straight antegrade intramedullary nail (SAIN) was either the anterolateral delta-split (group 2, *n* = 79) or the portal of Neviaser (group 3, *n* = 27). Length of surgery and time of radiation were extracted from charts. Patients stabilized using the PN were followed for a clinical and radiological exam. At follow-up we evaluated the DASH (Disability of the Arm, Shoulder and Hand) and CMS (Constant-Murley Score).

**Results:**

Between 10.2015 and 12.2018 191 proximal and diaphyseal humeral fractures were operated using either an angular stable extramedullary device (group 1, PHILOS®, *n* = 85) or a straight humeral nail (MultiLoc®, *n* = 106). Time of radiation and intervention followed a normal distribution. The mean length of surgery was 172.9 min (SD 91.5) in group 1, 121.5 min (SD 54.1) in group 2 and 96.4 min (SD 33.7) in group 3 (*p* < 0.01). Time of radiation was significantly different with 1.1 min (SD 0.6: group 1), 3.1 min (SD 1.6: group 2) and 2.9 min (SD 1.7: group 3) (p < 0.01). After a mean interval of 21.5 months (range 6–43 months) 14 / 27 patients of group 3 were available for a clinical and radiological follow-up. The mean DASH in group 3 was 25, the CMS reached 70. The age and sex weighted CMS mean value was 96%. Forward flexion was 131°, abduction 125°. The ratio of strength affected versus non-affected side was 4.4: 6.2 kg.

**Conclusions:**

The portal of Neviaser is a feasible and safe approach and is an alternative to the anterolateral delta-split. Length of surgery and time of radiation were significantly shorter.

**Level of evidence:**

IV

## Introduction

The use of a **s**traight **a**ntegrade **i**ntramedullary **n**ail (SAIN) using the delta-split approach is an established technique for the fixation of proximal and diaphyseal humeral fractures. However, the approach itself is frequently associated with pain and impaired shoulder function which is attributed to a damage to the hypovascular lateral zone of the rotator cuff [1]. Since any portal through the tendinous portion of the rotator cuff is regarded as potentially detrimental, alternative approaches are under continuous investigation.

A technical disadvantage of the delta split approach is a conflict with an overhanging acromion that can impede access to the starting point and might necessitate dissection of the coracoacromial ligament [2]. Furthermore, a too lateral nail entry might result into varus malalignment, which is an independent factor for loss of fixation [3].

It was therefore suggested to use a more medial approach located in the triangle between the posterior border of the clavicle and the acromion, the so called **P**ortal of **N**eviaser (PN) [4]. This approach was first used by Dilisio et al. in one patient with bilateral fractures and cuff arthropathy. The authors limited the indication to diaphyseal humeral fractures when access to the anterolateral starting port was not possible due to distorted shoulder anatomy [5]. The applicability was confirmed by Knierim et al. in 15 cadaver specimens. All nails passed entirely through the supraspinatus muscle belly [6].

It was therefore the objective of this study to evaluate the practicability of the PN compared to the delta-split approach in a clinical routine setting. Primary outcome parameters were length of surgery and radiation; secondary parameters were functional results using the DASH [7] and CMS [8].

## Materials and methods

### Patient selection

Patient data were retrospectively extracted from the electronic medical record of our hospital in a case series. All patients treated for humeral fractures between October 2015 and December 2018 were screened for eligibility. Inclusion criteria were fractures of the proximal, metaphyseal or diaphyseal humerus treated with an intramedullary device or an angular stable plate. Exclusion criteria were any precedent surgery. The analysis has been approved by the Comité National d’Ethique de Recherche of Luxembourg.

The MultiLoc® Humeral Nail and the PHILOS® (DePuy Synthes Companies, Switzerland) are standard implants for proximal and mid-shaft humeral fractures in our department. Their respective application is left to the surgeons` discretion. All patients admitted during the on-call of the first author were operated over the PN (*n* = 27), a bias concerning the complexity of the intervention can therefore be excluded. In the latter group, proximal fractures were classified according to the 12 categories described by Hertel et al. [9].

### Surgical technique

**Fig. 1:**
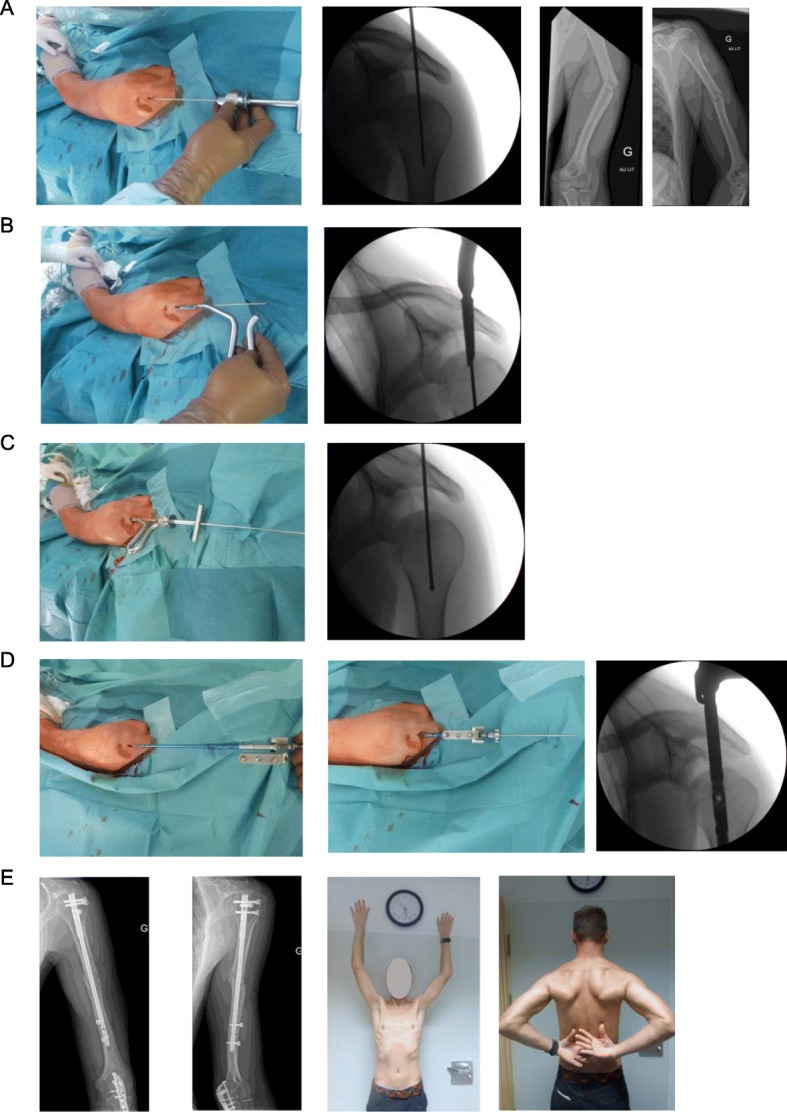
Combined diaphyseal and olecranon fracture. Superficial skin laceration next to the portal of Neviaser. A 1 cm skin-incision is made in the triangle between the clavicle, acromion and scapula spine. A Kirschner wire is advanced to the zenith of the humeral head (**a**). Once the entry point on the humeral head is established a canulated awl is advanced splitting the supraspinatus muscle fibres longitudinally and opening the the humeral head (**b**). With the awl in place the Kirschner wire is replaced by a longer guide (**c**) wire which is then used to advance the straight nail (**d**). Clinical and radiological follow-up 10 weeks after surgery (**e**)

**Fig. 2:**
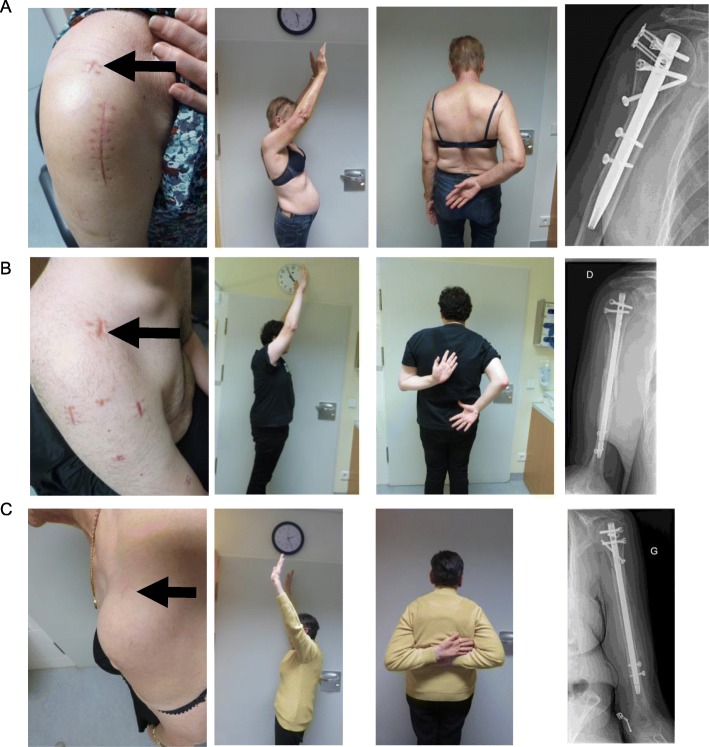
For nail insertion over Neviaser’s portal only a 1 cm incision is needed. Underlying soft-tissue structures are bluntly dissected. Skin incision is marked with a black arrow. D.C.P. female patient, 69 years. Three-part-fracture, an additional delta-split approach was necessary to reduce and stabilize an avulsed greater tubercle. Clinical and radiological follow-up at 8 months postoperative **a**. T.K. male patient, 24 years. Diaphyseal fracture, Clinical and radiological follow-up at 4 months postoperative **b**. U. female patient 66 years. Metaphyseal fracture. Clinical and radiological follow-up at 11 months postoperative **c**

**Fig. 3:**
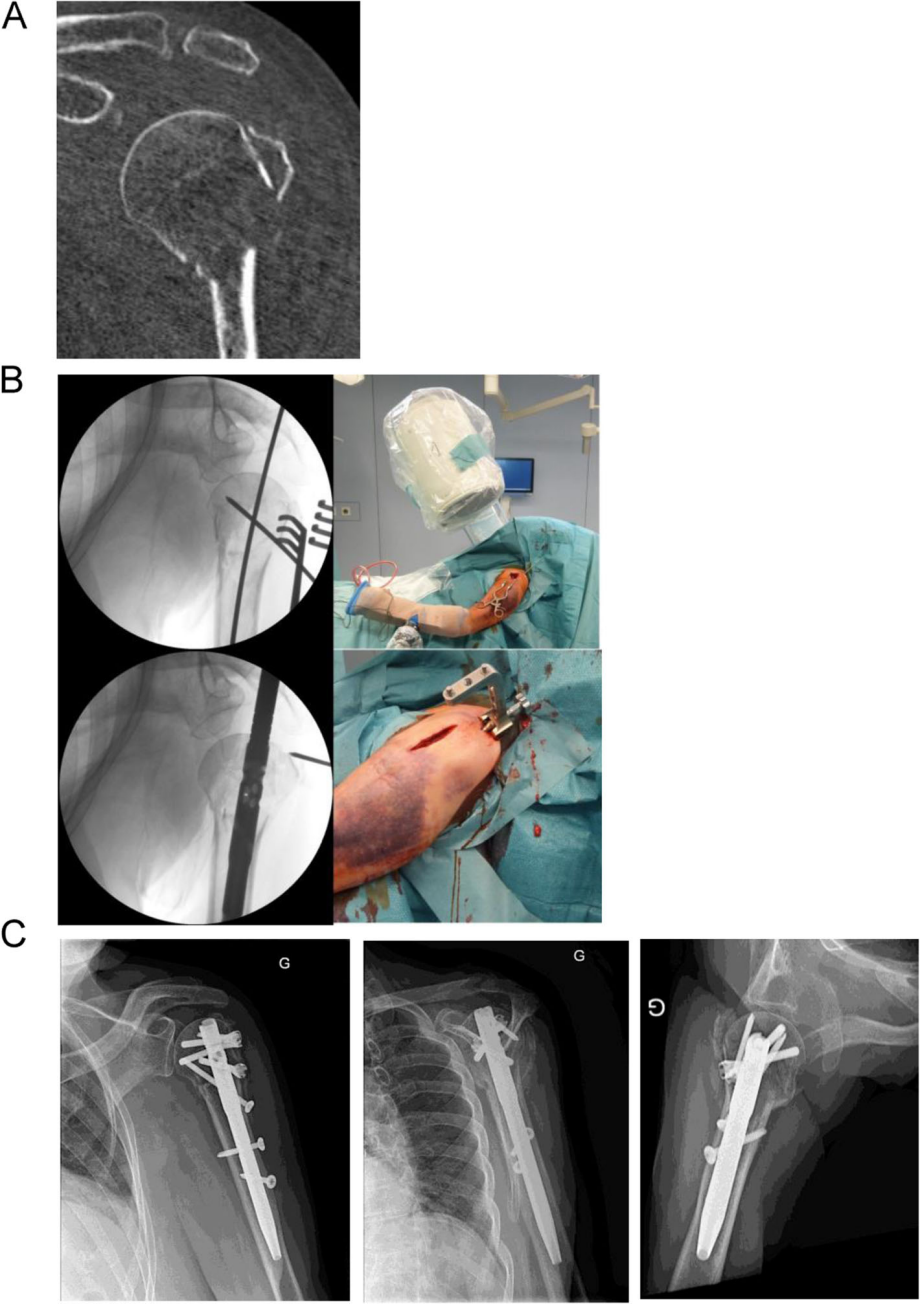
Female patient, 79 years. 3-part fracture of the proximal Humerus (**1a**). The anterolateral delta-split is used for fracture reduction using a Kirschner wire: introduction of the nail via PN. Confirmation of the appropriate depth of the nail (**1b**). Radiological follow-up at six weeks (**1c**)

**Fig. 4:**
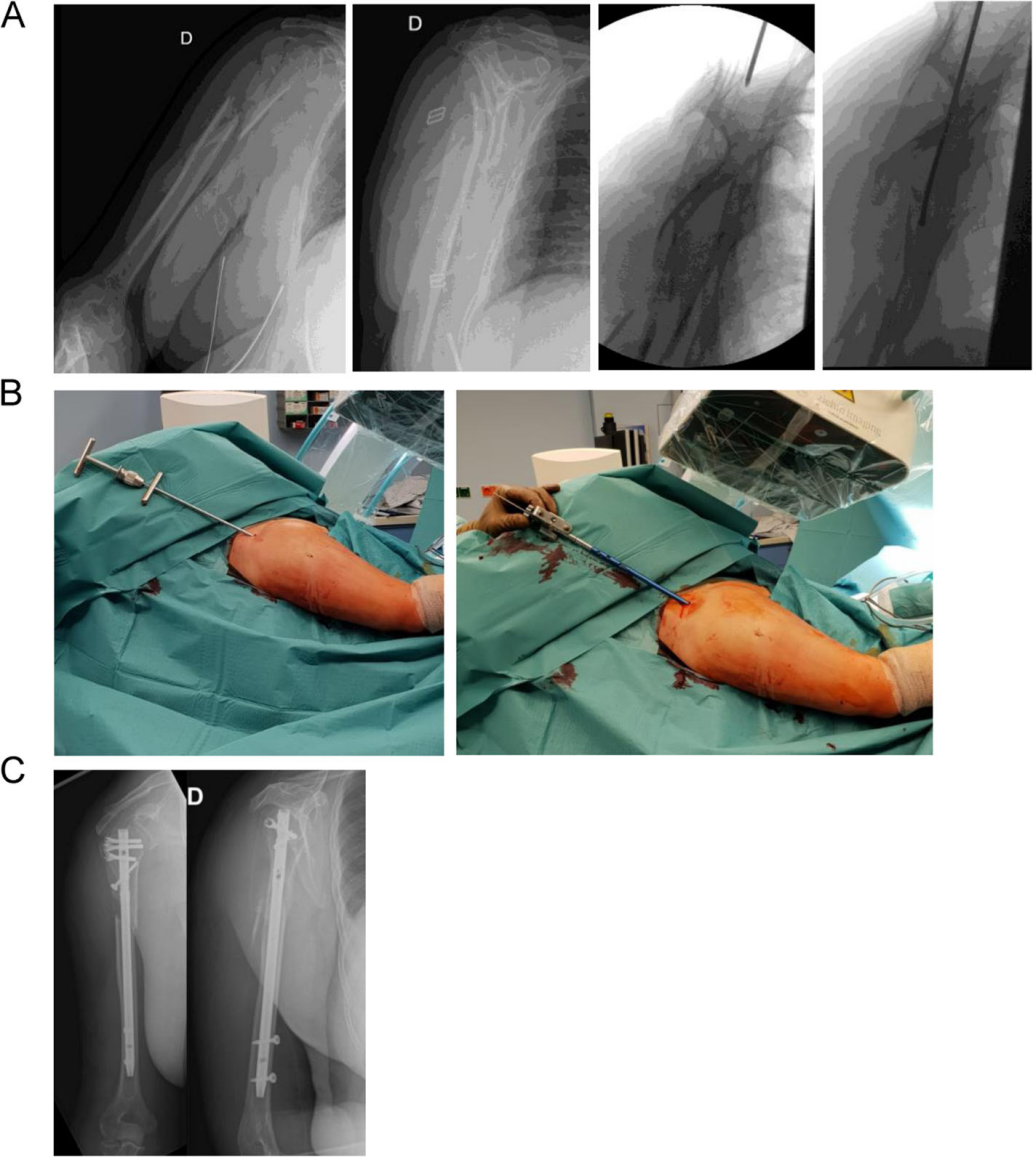
Female patient, 64 years. Metaphyseal fracture of the proximal Humerus. Rotating the image intensifier allows for ap and tangential view of the proximal humerus and determination of the entry point and direction (**2a**). Intraoperative view of the insertion site (**2b**) and immediate postoperative control (**2c**)

**Fig. 5:**
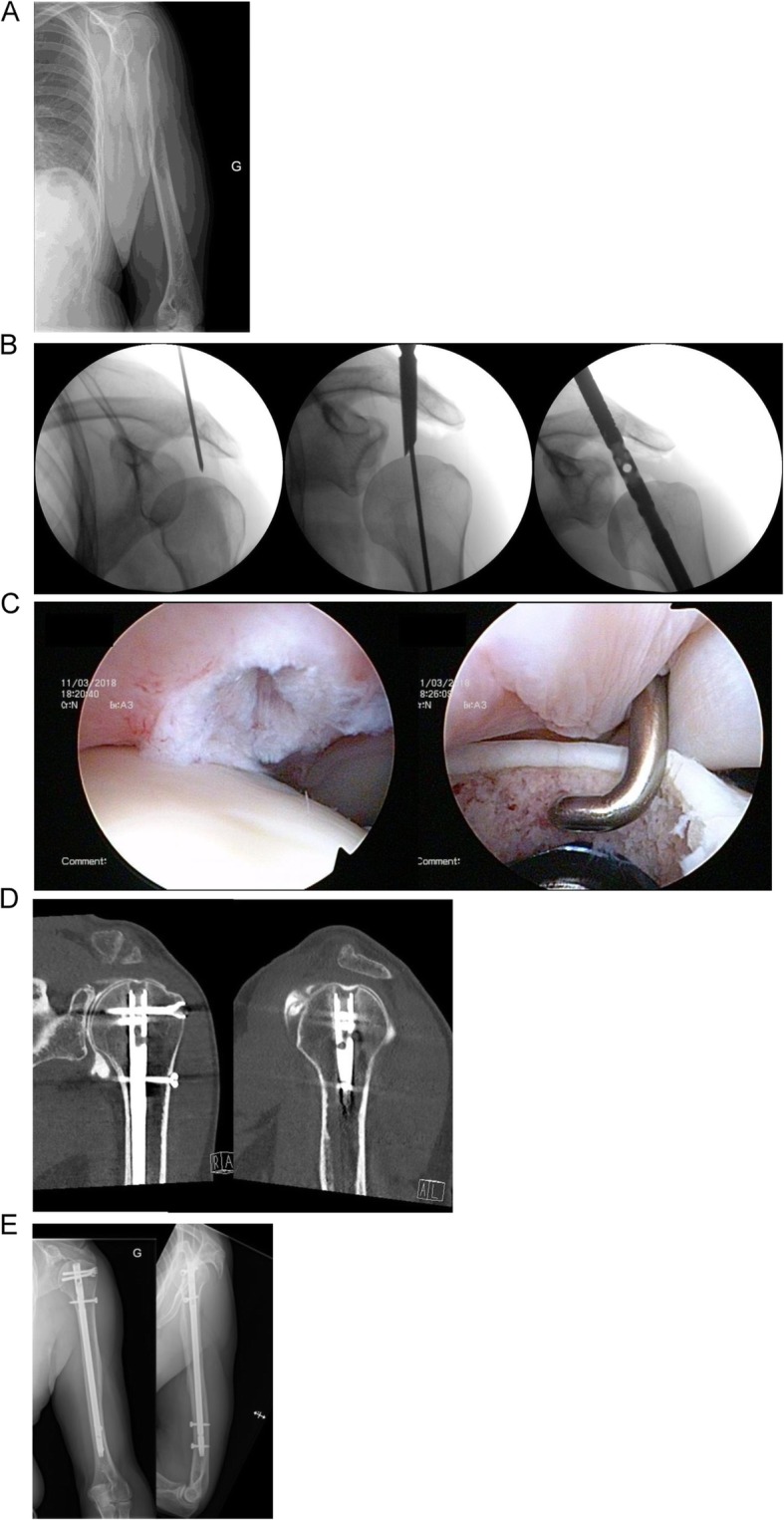
Male patient, 57 years, sustained a diaphyseal spiral fracture after a fall from stairs (**1a**). Three intraoperative views with the image intensifier: insertion point and direction was determined using a 2.0 mm Kirschner wire. After the feasibility has been verified a cannulated awl is used to perforate the supraspinatus tendon and the articular surface. With the awl in situ the Kirschner is removed and replaced by a long guide wire which then directs the nail (**1b**). After stabilisation a diagnostic arthroscopy is performed to document the perforation of the rotator cuff, the appropriate depth of the nail and to exclude concomitant intraarticular lesions (**1c**). Five months after surgery a CT with intraarticular application of contrast medium was performed; a leakage or lesion to the rotator cuff was excluded (**1d**). Bony consolidation at 6 months after surgery (**1e**)

Patients were operated in beach-chair position with the image intensifier on the opposite site. The forearm was fixed in the Trimano® (Arthrex Inc., Florida, USA) facilitating axial alignment by ligamentotaxis. The feasibility to use the PN was evaluated by a 2.0 mm Kirschner wire. The wire was introduced in the triangle between the dorsal border of the clavicle and the acromion, through the supraspinatus tendon and onto the humeral head. If the given entry point corresponded to the correct position and direction the Kirschner wire was further introduced. The skin incision was enlarged such that the supraspinatus tendon and the humeral head could be perforated with a cannulated awl. The Kirschner wire was than replaced with a guide wire. The MultiLoc® Humeral Nail was then introduced. Fixation of the nail was performed according to the recommendations of the manufacturer. (Fig. 1 a-e). The necessary skin incision was usually not larger than 1 cm (Fig. 2 a-c). If this did not result in a satisfactory reduction any additional percutaneous or open technique was applied as needed (Fig. 3 a-c). This surgical step of reduction was independent from the introduction of the nail. By rotating the image intensifier, the scapular Y-view confirmed the correct entry point in the second plane (Fig. 4 a-c). If indicated the osteosynthesis was combined with an arthroscopy (Fig. 5a-e). Physiotherapy was usually initiated on the first postoperative day.

### Outcome parameters

Length of surgery and time of radiation were recorded for all techniques. The systematic recall of patients was initiated between January and June 2019. At follow-up, patients stabilised using the PN were evaluated clinically to measure the range of motion and to calculate the DASH and CMS [10]. For measurement of muscle strength, the NorthStar Commander Echo from JTECH MEDICAL Industries (Midvale, Utah, USA) was used. At the same time radiological control was performed in standard projections. Depending on the clinical symptoms a CT scan was initiated.

### Statistical analyses

All statistical analyses were performed using SPSS version 24 (Statistical Package for Social Sciences, Chicago, IL, USA). *P-*values of < 0.05 were considered statistically significant. Descriptive statistics were used for analyzing patient demographics and clinical characteristics. Means and standard deviation (SD) were used for continuous variables, counts and percentages for categorical variables. Due to a wide distribution of scores, we also report median and min/ max scores. We used One-Way ANOVAs for the analyses on differences between the interventions. Levene’s test was used to test the assumption of equal variances. If violated, we used the Games-Howell post-hoc test, which does not assume equal variances.

## Results

Between Oct. 2015 and Dec. 2018, 85 fractures were stabilized using a PHILOS® (group 1), 106 fractures with a MultiLoc® Humeral Nail (group 2), whereof 27 were stabilized using the PN (group 3). The most frequent type of intracapsular fracture (*n* = 8) was type 7, a 3-part fracture, with a distal shaft fragment, fracture of the greater tubercle, and an intact fragment of head and lesser trochanter. Five patients had sustained a type 1, three patients a type 12, and eleven patients a diaphyseal fracture.

Most patients were female (69%); the average age was 67 years (SD 16) (Table 1). *A one-way between* subjects ANOVA was conducted to compare differences in age between the interventions. The intervention groups did not significantly differ according to age (n.s.) (Table 1). The median delay between hospitalisation and surgery was 3 days. Postoperatively, patients were seen at regular intervals after 6 weeks, 12 weeks, 6 months, and 1 year. A radiological follow-up was made on each of these consultations, independent of this analysis.

**Table 1:** Length of radiation and length of surgery in the different groups. * *p*-value < 0.01

	PHILOS Group 1	Delta Split Group 2	Portal of Neviaser Group 3
Age, mean (SD)	64 (16)	69 (13)	70.8 (18)
Radiation time min, mean (SD)*	1.1 (0.6)	3.1 (1.6)	2.9 (1.7)
Min, Max	.2, 2.2	1.3, 5.2	1.0, 5.4
Median	1.1	2.6	2.7
Intervention duration in min, mean (SD)*	172.9 (91.5)	121.5 (54.1)	96.4 (33.7)
Min, Max	63.0, 458.0	65.0, 246.10	53.0, 190.0

The average length of surgery was shortest for Neviaser with 96.4 min. (SD 33.7) and longest for PHILOS with 172.9 min. (SD 91.5). A one-way between subjects ANOVA was conducted to compare the differences in intervention duration between the interventions conditions. The intervention duration differed significantly between the different interventions [F (2, 53) = 7.132, (*p* = .002). The Levene’s test revealed heteroscedasticity. We therefore used the Games-Howell post-hoc test, which showed significant differences between Neviaser (group 3) and Philos (group 1; *p* = 0.001).

On average, the time of radiation was shortest for PHILOS (group 1) with 1.1 min. (SD 0.6) and longest for Delta Split (group 2) with 3.1 min. (SD 1.6). The one-way between subjects ANOVA on radiation time showed significant differences [F (2, 28) =8.927], (*p* = .001). The Levene’s test revealed heteroscedasticity. We therefore used the Games-Howell post-hoc test, which showed significant differences of radiation time between Neviaser (group 3) and Philos (group 1; *p* = 0.020).

In group 3, after a mean follow-up of 21.5 months 14 / 27 patients were available for a clinical and radiological follow-up; the mean value for the DASH was 25 (SD 26) (subjective 30, objective 40) and for the CMS 70 (SD 20) (Table 2).

**Table 2:** Functional results after stabilisation of humerus fractures using the portal of Neviaser

	Age at surgery	Age at follow-up	Follow-up	DASH	CMS	CMS age & sex weighted mean value	ROM Forward flexion	ROM Abduction	Strength (kg) of affected side	Strength (kg) of non-affected side
Minimum	28	31	6	0	24		74°	82°	0,5	3.5
Maximum	80	82	43	89	97.5		157°	154°	9.05	8.58
Range	52	51	37	89	73.5		83°	72°	8.55	5.08
Mean	63.5	65.07	21.5	25	70	96%	131	126	4.37	6.18
Standard Deviation	12.89	12.20	12.87	26	20		25	28	2.37	2.13
Confidence Intervall (95%)	6.75	6.39	6.74	14	11		13	15	1.24	1.21

After a mean interval of 21.5 months (range 6–43 months) 14 / 27 patients of group 3 were available for a clinical and radiological follow-up. 5 patients had deceased after a median of 13 months; 6 patients suffered from dementia or were non-compliant. 2 patients had moved from the area. The age and sex weighted mean value of the CMS demonstrated a functional outcome within the range of this specific population

Consolidation for proximal fractures was completed within the 3 months period after surgery and within the 6 months period for metaphyseal and diaphyseal fractures. No prolonged healing was observed

The aged & sex weighted mean value was 96%. Forward flexion was 131° (SD 25), abduction 125° (SD 28). The ratio of strength affected versus non-affected side was 4.4 kg (SD 2): 6.2 kg (SD 2).

## Discussion

The most important finding of this retrospective analysis was the observation that the portal of Neviaser is superior to the delta-split approach. It helps reducing time in the operating room and limits exposure to radiation.

The use of a **s**traight **a**ntegrade **i**ntramedullary **n**ail (SAIN) using the delta-split approach is an established technique for the fixation of proximal and diaphyseal humeral fractures. This straight design has advantages over a curvilinear design with regard to reoperation (42% versus 11.5%) and symptoms related to the rotator cuff (73% versus 34.6%) [1]. It is hypothesized that the persistent pain and compromised shoulder function is not only correlated with the implant but to the delta-split approach itself with damage to the critical hypovascular zone of the rotator cuff near its insertion on the humerus and/or irritation of the subacromial space [11].

Graticelli et al. performed an ultrasound exam after using a curvilinear nail design and observed partial ruptures of the rotator cuff in 32% and full-thickness ruptures in 13% [12]. Gierer et al. demonstrated a decrease of the functional capillar density of the supraspinatus tendon by 50% using a straight nail [13].

It is therefore advisable to choose a more medial approach through the supraspinatus muscle such as the portal of Neviaser. This is in line with the work of Boileau and Walch. According to their studies the entry point for a straight nail has to be close to the zenith in the ap and lateral projection [14]. At the same time it is known that 38.5% of the humeral heads can be categorised as ‘critical types’, meaning that the predicted offset of the entry point will encroach on the insertion of the supraspinatus tendon [15]. This zone can be regarded as vulnerable with a limited healing response. Brooks et al. demonstrated in a histological study that most vessels run in the long axis of the tendon with a poor filling in the distal 15 mm. Additionally, number and diameter of vessels decrease towards the humeral insertion [16].

To circumvent this problem Park et al. described an approach utilizing the rotator cuff interval; they followed 33 patients with 34 humeral fractures with an average of 34 months. The overall satisfaction rate was more than 90%, according to the ASES (American Shoulder and Elbow Society) score. The mean Constant-Murley-Score was 84 (SD, 14; range, 17 to 98), and primary bone union was achieved in 32 of the 34 cases [17]. Later, this technique was combined with a biceps tenodesis [11]. It was thought that this approach allows access to the optimal humeral nail starting point while potentially decreasing postoperative shoulder pain. Still, this incision affects the lateral hypovascular zone of the rotator cuff.

Because of the limitations of current surgical techniques we adapted the technique introduced by Dilisio [5] and Knierim et al. [6]. Using the portal of Neviaser the problem of a lateral entry point that puts the supraspinatus tendon at risk was circumvented. At the same time the direction of the guide wire and nail were aligned with the humeral shaft axis. Any iatrogenic varus reduction could hereby be avoided. The strict percuteanous technique reduced surgical time and exposure to radiation. The clinical follow-up demonstrated outcome parameters such as DASH and CMS within the range of a recent meta-analysis by Wong et al. [18]. They included 14 studies with a mean follow-up of 22.6 months. The overall frequency-weighted mean CMS was 72.8. Frequency-weighted mean forward flexion and abduction were 137.3°, 138.4°, respectively.

## Limitations

Any conclusion is limited by the rather small number of patients treated and the small number available for follow-up. A further evaluation necessitates a prospective-randomized approach; in the view of the age and frailty of the patients preferably in a multi-center design.

Using the delta-split approach the supraspinatus tendon is repaired under visual control; this is not necessary using the portal of Neviaser since the cutaneous incision is no longer than 1–2 cm and medially located such that it does not affect the tendinous portion. The long-term effect of the incision in the musculo-tendinous junction is unknown and needs further evaluation by ultrasound or MRI.

A technical aspect is the instrumentation; this was designed for an open approach. For the minimal-invasive approach it needs to be modified using a longer introductory handle, comparable to the suprapatellar nailing of the tibia.

## Conclusion

Twentyseven patients with fractures of the proximal humerus and the humeral shaft were stabilized by antegrade nailing over the portal of Neviaser and followed prospectively. We could demonstrate that length of surgery and exposure to radiation were reduced compared to the delta-split approach. DASH, CMS and range of motion were comparable to data from a recent meta-analysis.

## Abbreviations

ASES: American Shoulder and Elbow Society [18]
CMS: Constant-Murley-Score
DASH: Disability of the Arm, Shoulder and Hand [7]
PN: Portal of Neviaser
ROM: Range of Motion
SAIN: straight antegrade intramedullary nail (−ing)
SD: Standard Deviation
